# Longitudinal associations between medication use and phenotypic aging: insights from the Baltimore longitudinal study of aging

**DOI:** 10.1093/gerona/glaf128

**Published:** 2025-06-11

**Authors:** Bowen Tang, Perry Kuo, Ann Zenobia Moore, Madhav Thambisetty, Luigi Ferrucci, Sara Hägg

**Affiliations:** Department of Medical Epidemiology and Biostatistics, Karolinska Institutet, Stockholm, Sweden; Brain Aging and Behavior Section, National Institute on Aging, Baltimore, Maryland, United States; Longitudinal Studies Section, National Institute on Aging, Baltimore, Maryland, United States; Longitudinal Studies Section, National Institute on Aging, Baltimore, Maryland, United States; Longitudinal Studies Section, National Institute on Aging, Baltimore, Maryland, United States; Brain Aging and Behavior Section, National Institute on Aging, Baltimore, Maryland, United States; Longitudinal Studies Section, National Institute on Aging, Baltimore, Maryland, United States; Department of Medical Epidemiology and Biostatistics, Karolinska Institutet, Stockholm, Sweden

**Keywords:** medications, phenotypic aging, longitudinal study

## Abstract

**Background:**

Limited population-based data exist on the association between medication use and changes in phenotypic aging. This study investigated these associations using data from the Baltimore Longitudinal Study of Aging.

**Methods:**

Phenotypic aging (PA) markers were constructed using the Klemera-Doubal method across four domains: body composition (structural and metabolic changes), energetics (energy generation and utilization capacity), homeostatic mechanisms (internal stability maintenance), and neuroplasticity/neurodegeneration (nervous system function and decline). Associations between 27 common drug categories and changes in these PA markers were analyzed using conditional generalized estimating equations (cGEE), focusing on within-individual variation to control for genetics and early-life factors, with additional adjustments for time-varying covariates.

**Results:**

Five drug categories were associated with significant reductions in PA markers. Vitamin D, bisphosphonates, and proton pump inhibitors were linked to decreases in body composition (Beta = −0.73 years, 95% CI: −1.35 to −0.10), energetics (Beta = −2.05, 95% CI: −3.98 to −0.13), and neuroplasticity/neurodegeneration (Beta = −1.00, 95% CI: −2.02 to −0.03), respectively. Thyroid hormones showed reductions in body composition (Beta = −1.75, 95% CI: −3.24 to −0.26) and neuroplasticity/neurodegeneration (Beta = −1.04, 95% CI: −1.96 to −0.12). Thiazides were associated with decreases across body composition (Beta = −1.55, 95% CI: −2.94 to −0.16), energetics (Beta = −2.36, 95% CI: −4.30 to −0.42), and homeostatic mechanisms (Beta = −3.83, 95% CI: −6.71 to −0.96).

**Conclusions:**

These findings suggest potential protective effects of certain medications on phenotypic aging. Further research is needed to validate these results, particularly with data from other populations.

## Introduction

Aging is a complex biological process marked by the gradual decline of physiological functions and increased susceptibility to diseases and mortality.^1^ Geroscience views aging as a major driver of chronic diseases like cardiovascular and neurodegenerative disorders. Targeting the biological mechanisms of aging to slow or reverse its effects offers a promising strategy to prevent or delay most chronic diseases, extend lifespan, and improve healthspan.^2^ While over 400 compounds have shown potential in extending lifespan in model organisms, translating these findings to humans is challenging due to the cost and duration of human longevity studies.^3^ A more feasible approach in human research involves the use of biological age biomarkers, such as telomere length, epigenetic clocks, and composite indices, that capture the biological aging process and predict lifespan, serving as surrogate endpoints for anti-aging interventions.^4^ These BA biomarkers, shown to be modifiable in some clinical trials, have correlated with improvements in biological systems, such as reduced senescent immune cells and better immunological profiles, supporting their potential to guide the development of effective anti-aging therapies.^5^

Over the past decade, the “hallmarks of aging” framework has become a foundational concept in geroscience, outlining 12 key processes that operate at the molecular and cellular levels.^6^ Accumulated disturbances in these hallmarks manifest as changes in physiological and anatomical functions. Aging, therefore, arises from a complex interplay of molecular and cellular mechanisms that ultimately result in phenotypic changes across multiple physiological systems.^7^ To better quantify aging, it is essential to examine phenotypic aging (PA).^8^ This concept has been refined within the Baltimore Longitudinal Study of Aging (BLSA), which identified four critical domains of phenotypic aging: body composition (structural and metabolic changes), energetics (capacity to generate and utilize energy), homeostatic mechanisms (maintenance of internal stability), and neuroplasticity/neurodegeneration (nervous system function and deterioration).^9^

Despite these advances, population-based data on the relationship between medication use and changes in aging process remain scarce.^10^^,^^11^ Longitudinal studies, such as the BLSA—where individuals participate in repeated assessments of phenotypic aging markers as well as collection of information on their usual life course exposures including pharmaceutical use—provide a robust framework for evaluating how pharmaceutical interventions might influence the aging process.^12^^,^^13^ In this study, we aimed to evaluate the effects of commonly used medications on phenotypic aging by applying a within-individual statistical approach to BLSA data. This method, which is similar to a self-controlled design, compares periods of drug use to periods of non-use within the same individual, thereby controlling for time-invariant factors such as genetics, sex, and race.^14^

## Methods

### Study design and population

Initiated in 1958, the BLSA was significantly updated in 2003 to include comprehensive phenotypic measurements across four domains: body composition, energetics, homeostatic mechanisms, and neuroplasticity/neurodegeneration.^13^ These domains bridge geroscience, which focuses on cellular and molecular mechanisms of aging, with gerontology and geriatrics, which study age-related diseases and functional decline.^9^ The BLSA is a continuously enrolled cohort study of community-dwelling U.S. residents who are free of significant chronic conditions at their first study visit. Participants are followed at intervals determined by age: every four years for those under 60, every two years for those aged 60-79, and annually for those aged 80 and older.

**Figure 1.**
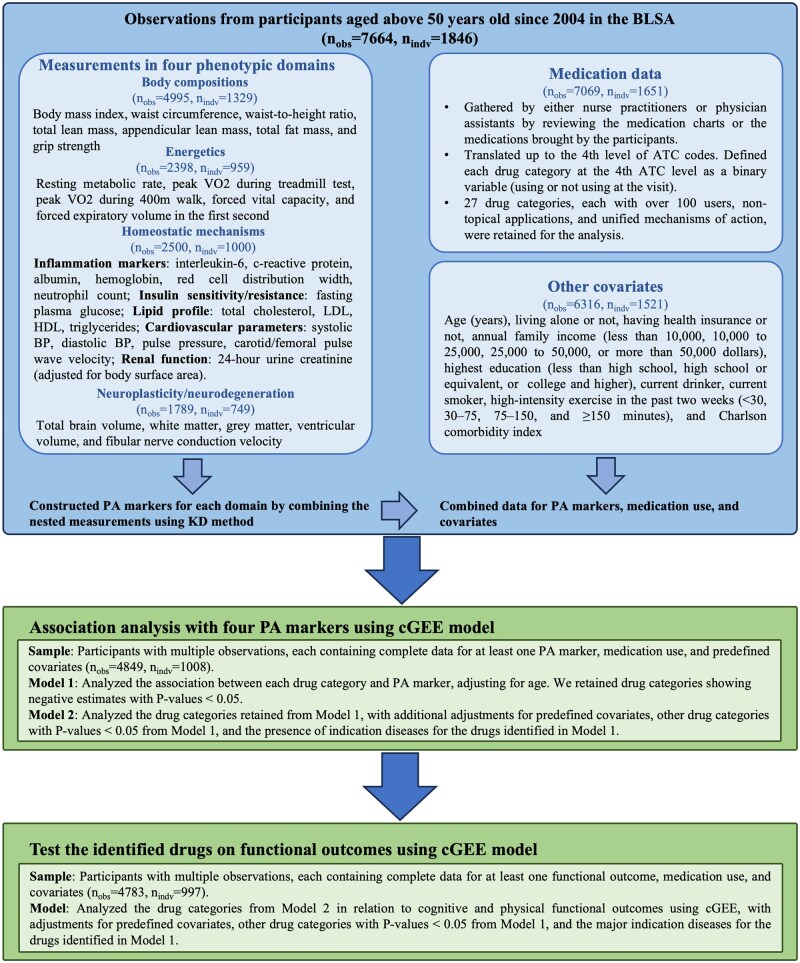
Study design and inclusion criteria for the current study. n_obs_ refers to the number of longitudinal observations, and n_idv_ represents the number of unique participants contributing to those observations. KD method was used to estimate phenotypic aging (PA) makers. The analysis employed cGEE (conditional generalized estimating equations) to account for individual-constant factors over time. ATC, Anatomical Therapeutic Chemical codes; BLSA, Baltimore Longitudinal Study of Aging; BP, blood pressure; HDL, high-density lipoprotein; KD, Klemera–Doubal method; LDL, low-density lipoprotein; VO2, oxygen consumption.

For this study, we analyzed data from participants who underwent phenotypic measurements after 2004, focusing on older individuals aged 50 and above to account for the non-linear changes in some phenotypic measurements that occur from young to older age. We constructed a single PA marker for each phenotypic domain using complete data from the respective domain’s measurements. We then examined the associations between various medications and changes in these four PA markers. Participants with multiple observations and complete data for at least one PA marker, medication use, and essential covariates were included in the study sample; the analyses included 4 894 observations from 1 008 participants. Cognitive and physical performance measurements were also obtained to assess whether the identified medications influenced functional outcomes. Figure 1 outlines the study design and inclusion criteria.

### Phenotypic aging measurements

The biomarkers nested within the four phenotypic domains—body composition, energetics, homeostatic mechanisms, and neuroplasticity/neurodegeneration—are presented in Figure 1. The details of these measurements are described in other papers.^15^^,^^16^ For each domain, we constructed a PA marker using the complete dataset of the nested phenotypic measurements (see Statistical Methods). We excluded extreme values separately for men and women within each age group (50-90 years old in 10-year increments, and over 90) using Tukey's method, aiming to eliminate outliers that might have resulted from measurement errors or severe conditions. Log transformations were applied to normalize variables with skewed distributions. Figures S1-S4 illustrate the age-related trajectories of these phenotypic measurements, mostly demonstrating an approximate linear change.

In addition to the four PA markers, we included PhenoAge, a widely-used aging biomarker developed by Levine et al., to evaluate its associations with the identified medications.^17^ The nine biomarkers described by Levine et al., with their trajectories presented in Figure S5, were extracted from the BLSA dataset and integrated using the algorithms implemented in the BioAge R package to calculate PhenoAge separately for men and women in our study participants.^18^ Cognitive and physical functional measurements were explored to determine whether identified medications could benefit functional outcomes. Physical function was represented by the Health, Aging and Body Composition short physical performance battery (HABC SPPB), a continuous score derived from four measurements: usual gait speed; time taken to stand up and sit back down five times on an armless chair without assistance; ability to hold three balance-related positions (semi-tandem, full-tandem, and single-leg stands) for up to 30 seconds each; and capacity and time to walk a narrow (20 cm wide) 6-meter course.^19^ Global cognitive function was assessed using the Mini-Mental State Examination (MMSE).^20^

### Medication use and other covariates

Medication use was documented at each visit by nurse practitioners or physician assistants, who reviewed participants’ medication charts or brought medications.^21^ Medications were classified using the Anatomical Therapeutic Chemical (ATC) coding system at the 4th level, and drug use was recorded as a binary variable (use vs. non-use) at the visit. We restricted our analysis to systemic drugs used by at least 100 participants, resulting in 27 drug categories. We excluded drug categories with topical applications or combinations of multiple active ingredients (e.g., vitamin A and D combined) because it would be difficult to identify which specific compound contributes to changes in PA markers, making the interpretation of individual drug effects less clear.

We included time-varying factors, including age, living alone, health insurance status, annual family income (<$10 000, $10 000-$25 000, $25 000-$50 000, and ≥$50 000), highest education level (less than high school, high school graduate, some college, college graduate, and post-college), current smoking and drinking status, high-intensity exercise in the past two weeks (<30, 30-75, 75-150, and ≥150 minutes), and Charlson Comorbidity Index (CCI), as covariates. These data were collected during comprehensive interviews at each visit. Missing covariate data were imputed for the same individual using the most recent historical non-missing data within a 5-year window.

### Statistical methods

First, we constructed a PA marker for each of the four phenotypic domains (body composition, energetics, homeostatic mechanisms, and neuroplasticity/neurodegeneration) by integrating the nested measurements using the Klemera-Doubal (KD) method. The KD method estimates biological age by calculating a weighted average of marker deviations from age-based norms. Each biomarker is weighted by its association with chronological age (CA), allowing the method to capture individual differences from the expected age profiles in a reference sample.^22^ Before applying the KD method, several adjustments were made to specific measurements. Energetic measurements were adjusted for BMI or height. Laboratory measurements for homeostatic mechanisms were adjusted for measurement techniques, and brain volumes for the neuroplasticity/neurodegeneration were adjusted for MRI scanners and intracranial volume.^15^

We performed 10 iterations of 10-fold cross-validation to estimate the PA marker for each domain, analyzing men and women separately. For each iteration, one set was used as the test set, while the remaining nine were used for training. Participants were randomly shuffled and divided to create the training/testing splits.^23^ To maintain independence, the training sets included only the first available observation per participant. All measurements were z-score normalized based on the mean and standard deviation of the training set for each fold. Principal components (PCs) were calculated from the training sets and then applied to all samples to ensure linear independence. The derived PCs were used as input for the KD method to estimate the PA marker. Age was not included, as doing so would reduce the contribution of the aging phenotypes in estimating the PA marker.^24^ The PA markers for participants in the test sets were predicted using the parameters estimated from the training sets. For each domain, the predictions from the ten iterations were averaged. We described the trajectory of the four PA markers across age using a linear mixed model, with the interaction term between age and sex as the fixed effect and individuals as random effects. The correlation between the PA markers and age was assessed using the repeated measures correlation method.^25^

To validate the PA markers as predictors of aging, we examined their association with mortality risk. Vital status was determined through telephone follow-ups, correspondence, and National Death Index searches, with follow-up continuing until the last known status date.^16^ The association between each PA marker and mortality risk was assessed using Cox models with the follow-up time as time scale, adjusting for CA and sex. The first available observation with a non-missing PA marker value was used for survival analysis.

We used conditional generalized estimating equation (cGEE) models to estimate the associations between medication use and changes in PA markers. This model focuses on within-individual variation by conditioning on individuals and incorporating an individual-specific intercept. This approach allows for controlling individual-constant factors, enabling the examination of how changes in medication use are associated with changes in PA markers over time within the same individuals.^11^^,^^14^^,^^26^ For each PA marker, a cGEE model was constructed with medication use as the exposure and the PA marker as the outcome. Medications are often prescribed in response to disease onset or progression that likely accelerates the aging process. Consequently, positive associations between medications and PA markers could indicate disease progression rather than drug effects. To mitigate such potential false positives, we focused on medications with negative estimates, suggesting possible protective effects on aging, even in the presence of indication bias. We employed a 2-step strategy for association testing: Model 1: Adjusted only for age to identify drugs showing any negative association with the PA marker. Model 2: Included additional adjustments for time-varying covariates, other drug categories with a *P *< .05 from Model 1 (shown in Table S1), and the major indication diseases for drugs retained from Model 1. We also conducted subgroup analyses for these drugs in Model 2, stratifying by sex (men and women) and race (White and African American). Other racial groups were excluded due to limited sample sizes. Additionally, we examined associations between identified drugs and PhenoAge as well as cognitive and functional outcomes. For this exploratory study, we used a significance threshold of *P*-value < .05. All analyses were performed using R version 4.4.0.

## Results

### Trajectories of PA markers across four domains

We included 4 995 observations for constructing the PA marker for body composition (n = 1 329 individuals), 2 398 observations for energetics (n = 959), 2 500 observations for homeostatic mechanisms (n = 1 000), and 1 789 observations for neuroplasticity/neurodegeneration (n = 749).

**Figure 2.**
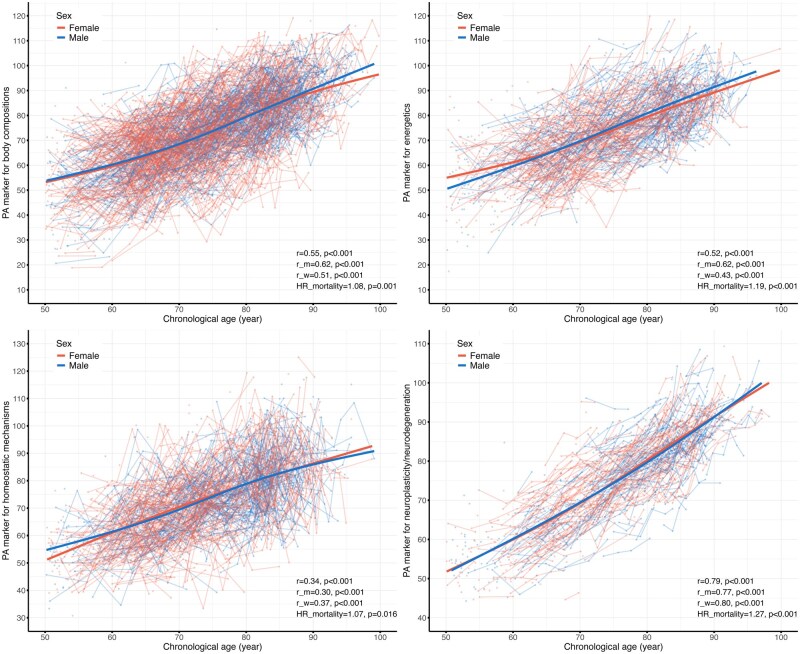
Trajectories of phenotypic aging markers for body composition, energetics, homeostatic mechanisms, and neuroplasticity/neurodegeneration over age. Each dot represents the estimated phenotypic age (PA) marker for a single observation, while the spaghetti lines illustrate changes between observations for the same participant. The trajectories were estimated separately for men and women using a linear mixed model, with an interaction term of age and sex as the fixed effect, and individuals as the random effect. The r represents the correlation coefficient, estimated using the repeated measures correlation method. The correlation coefficients are provided for the overall sample (r), men (r_m), and women (r_w). The HR_mortality represents the hazard ratio per a five-year increase in the PA marker from a Cox model, adjusted for age and sex. The first available observation for each participant with a non-missing PA value was used in the survival analysis. HR, hazard ratio.

The trajectories of the four PA markers are illustrated in Figure 2. All four PA markers demonstrated an increase with age, though the strength of their correlations with age varied, with coefficients ranging from 0.34 to 0.79 (all *P *< .001). Additionally, all four PA markers independently predicted mortality, with hazard ratios (HRs) for mortality, adjusted for age, ranging from 1.07 to 1.27 per 5-year increase in the PA marker (all *P *< .05, Figure 2 and Table S2). In comparison, PhenoAge showed a more pronounced increase with age (correlation coefficient = 0.92, *P *< .001) and demonstrated a stronger association with mortality (HR = 1.36 per 5-year increase in PhenoAge, *P *< .001) (Figure S6). The correlation between the four PA markers was mild to moderate, with coefficients ranging from 0.16 to 0.47 (*P *< .05). These correlations attenuated to null after adjusting for the effects of age (coefficients from −0.04 to 0.07, *P *< .05), except between body composition and energetics (coefficient = 0.14, *P *< .05, Figure S7).

### Association between medication use and PA markers

The characteristics of the participants included in the association analysis are described at their first and last available observations (Table 1). Over the follow-up period, there was a significant decline in the proportion of participants reporting current alcohol consumption (*P *< .05) and participation in high-intensity exercise (*P *< .001). Additionally, the proportion of participants with higher family incomes showed a marginal decrease (*P *= .059). Moreover, the number of participants living alone increased (*P *= .007), as did the average Charlson Comorbidity Index (CCI) (*P *= .069).

**Table 1.** Characteristics of participants included in the analysis of associations between medication use and changes in four phenotypic aging markers.

	First available visit	Last available visit	*P*-value
**No. of participants**	1008		
	**Individual-constant variables**		
**Sex = Male (%)**	466 (46.2)		
**Race (%)**			
White	696 (69.0)		
Black or African American	254 (25.2)		
Asian	40 (4.0)		
Other race	18 (1.8)		
	**Time-varying variables**		
**Age (mean [SD], years)**	69.68 (10.48)	78.62 (9.49)	<.001
**Current smoker = Yes (%)**	21 (2.1)	18 (1.8)	.75
**Current drinker = Yes (%)**	840 (83.3)	787 (78.1)	.003
**High-intensity exercise in the past 2 weeks**			<.001
Less than 30 minutes	315 (31.2)	450 (44.6)	
Between 30 and 75 minutes	122 (12.1)	120 (11.9)	
Between 75 and 150 minutes	157 (15.6)	129 (12.8)	
More than 150 minutes	414 (41.1)	309 (30.7)	
**Having health insurance = Yes (%)**	994 (98.6)	1000 (99.2)	.28
**Highest education (%)**			.43
Less than high school			.66
High school	7 (0.7)	6 (0.6)	
Some college	109 (10.8)	92 (9.1)	
College grad	93 (9.2)	99 (9.8)	
Post college	287 (28.5)	276 (27.4)	
**Household income per year (%)**			.059
Less than $10 000	10 (1.0)	18 (1.8)	
10000 to 25 000	40 (4.0)	61 (6.1)	
25000 to 50 000	174 (17.3)	159 (15.8)	
More than 50 000	784 (77.8)	770 (76.4)	
**Living alone = Yes (%)**	263 (26.1)	319 (31.6)	.007
**CCI (mean [SD])**	0.96 (1.56)	1.09 (1.66)	.067

Abbreviation: CCI, Charleston Comorbidity Index. Participants with multiple observations, each containing complete data for at least one phenotypic aging marker, medication use, and predefined covariates, were included in the analysis. The first and last visit were the first and last one available in the final retained dataset. Individual-constant variables refer to factors that remain unchanged for the same individual over time, whereas time-varying variables are those that fluctuate and were included as covariates for adjustment.

We tested 27 drug categories for their association with each of the four PA markers in Model 1, adjusting only for age using the cGEE model (see overall results in Table S1). Table 2 presents the results for five drug categories with *P *< .05 for a negative association with at least one PA marker: vitamin D and analogues, proton pump inhibitors (PPIs), bisphosphonates, thyroid hormones, and thiazides. We further examined these statistically significant medication-PA marker associations in Model 2, which adjusted for predefined time-varying covariates, other drug categories with *P *< .05 from Model 1 (Table S1), and presence of indication diseases for the five identified drug categories including hypertension, dyslipidemia, heart failure, myocardial infarction, edema, hypothyroidism, vitamin D deficiency, hypocalcemia, osteoporosis, and diseases of the esophagus, stomach, and duodenum.

**Table 2.** Associations between medication use and markers across four phenotypic domains.

In Model 2, the five drug categories maintained associations with decreases in the PA markers (Table 2). Vitamin D and analogues were associated with a decrease in the PA marker for body composition (Beta = −0.73 years, 95% CI = −1.35 to −0.10, *P *= .023). PPIs were linked to a reduction in the PA marker for neuroplasticity/neurodegeneration (Beta = −1.00, 95% CI = −2.02 to 0.03, *P *= .056), though it became marginally significant. Bisphosphonates showed an association with a decrease in the PA marker for energetics (Beta = −2.05, 95% CI = −3.98 to −0.13, *P *= .037). Notably, thyroid hormones and thiazides were associated with decreases in multiple PA markers. Thyroid hormones showed associations with PA marker for body composition (Beta = −1.75, 95% CI = −3.24 to −0.26, *P *= .022) and neuroplasticity/neurodegeneration (Beta = −1.04, 95% CI = −1.96 to −0.12, *P *= .027). Thiazides demonstrated associations with PA marker for body composition (Beta = −1.55, 95% CI = −2.94 to −0.16, *P *= .028), energetics (Beta = −2.36, 95% CI = −4.30 to −0.42, *P *= .017), and homeostatic mechanisms (Beta = −3.83, 95% CI = −6.71 to −0.96, *P *= .0088). Most of these results were consistent in subgroup analyses by sex (women and men) and race (White and African American), although significance often attenuated due to smaller sample sizes in stratified analyses (Table S3).

### Associations with PhenoAge and cognitive and physical function outcomes

When examining the associations of identified drugs with PhenoAge as well as cognitive and physical function outcomes in fully adjusted models, we found that Vitamin D and its analogues were associated with a significant reduction in PhenoAge (Beta = −0.35, 95% CI = −0.59 to −0.12, *P *= .0033). No associations were observed for the other four drug categories, although bisphosphonates (Beta = −0.14, 95% CI = −0.53 to 0.26, *P *= .49) and thiazides (Beta = −0.52, 95% CI = -1.14 to 0.10, *P *= .10) showed possible trends toward negative associations (Table S4). Regarding cognitive and physical function outcomes, we found that PPIs were associated with subtle improvements in cognitive function, as measured by MMSE (Beta = 0.17, 95% CI = 0.01 to 0.33, *P *= .03) (Table S4). No association was observed for the other drug categories.

## Discussion

In this study, we investigated the associations between medication use and changes in aging biomarkers across four key phenotypic domains—body composition, energetics, homeostatic mechanisms, and neuroplasticity/neurodegeneration—using longitudinal data from over 1 000 participants in the BLSA. Of the 27 drug categories examined, five—vitamin D and analogues, PPIs, bisphosphonates, thyroid hormones, and thiazides—were associated with decreases in one or more PA markers after adjusting for key covariates and indication diseases.

Several population-based studies have investigated the effects of drugs on markers of aging, but they have primarily focused on biomarkers at the molecular level, such as epigenetic clocks, or at the functional level, using indices like the functional age index.^10^^,^^11^^,^^27^^,^^28^ Our study, however, adopts a novel approach by examining multidomain phenotypic aging. Research from the BLSA has demonstrated that accelerated phenotypic aging across four domains is linked to faster declines in physical and cognitive function, increased multimorbidity, and reduced life expectancy.^16^ Our findings further revealed that all PA markers constructed from the four phenotypic domains independently predict a higher mortality risk, even after controlling for CA. However, compared with PhenoAge the four PA markers demonstrated weaker correlations with CA and smaller associations with mortality. This difference might be explained by the fact that PhenoAge was trained specifically to predict aging-related mortality and explicitly includes CA as a component in its construction.^17^ In contrast, our approach employed a modified KDM designed to better reflect biological aspects of aging by limiting the influence of CA in the calculation.^24^ Interestingly, except for the body composition and energetics domains, the correlations between these PA markers shifted from moderate to negligible after adjusting for chronological age. This attenuation suggests that while these markers capture some common aging processes, they primarily reflect domain-specific mechanisms of phenotypic aging. Collectively, these findings from the BLSA highlight the importance of a multidimensional phenotypic perspective on aging, which could help identify individuals at risk for accelerated aging and inform the development of interventions targeting both cellular damage and broader physiological declines.

In this study, five drug categories were associated with decreases in one or more PA markers across the four domains. Vitamin D and bisphosphonates, commonly used in older populations to maintain or improve bone density in conditions like osteoporosis,^29^^,^^30^ have shown favorable effects on aging, such as reductions in epigenetic clocks and all-cause mortality risk, in clinical trials.^31–33^ The effects of thiazides—a type of diuretic primarily used to treat hypertension—on aging are relatively understudied, and the impact of PPIs, which are acid inhibitors used for treating gastrointestinal conditions, especially on neurodegeneration, remains contentious.^34^ For instance, research on PPI use and dementia risk has produced conflicting results, with some studies suggesting an increased risk, while others found no significant association.^35–38^ Therefore, our finding of an association between PPI use and reduced PA markers of neuroplasticity/neurodegeneration should be interpreted with great caution. Interestingly, thiazides have been shown to increase bone density and reduce fracture risk in older adults, likely through mechanisms such as reduced renal calcium excretion and modulation of bone turnover.^39^ These beneficial effects on bone health, shared by vitamin D, bisphosphonates, and thiazides, may partly explain their associations with reduced aging in body composition and energetics, underscoring the importance of bone health in the aging process of older adults. Thyroid hormone replacements were associated with reductions in PA marker for both body composition and neuroplasticity/neurodegeneration. Given that these hormones are commonly used to treat hypothyroidism, a condition that can impair cognitive function in older adults, the observed reductions in the neurodegeneration biomarker may reflect the alleviation of hypothyroidism-related cognitive deficits. However, research indicates that while thyroid hormone therapy can improve symptoms of hypothyroidism, it may not fully reverse cognitive decline.^40^ Moreover, excessive levels of thyroid hormones, as seen in hyperthyroidism, have also been linked to cognitive dysfunction.^41^ Therefore, the relationship between thyroid hormone levels, replacement therapies, and their effects on neurodegeneration and brain aging is complex and warrants further investigation. Indeed, many of the drugs identified in our study exhibit potential pleiotropic effects in addition to their approved uses.^42–46^ Our study is the first to propose hypotheses suggesting that drugs like thiazides may have beneficial effects on aging. However, it is important to emphasize the exploratory nature of this research, and further validation is necessary to confirm these findings.

In our study, we observed a significant association between vitamin D use and a reduction in PhenoAge, while no such associations were found for the other four drug classes. Importantly, no medication was consistently associated with all the assessed aging biomarkers in the current study. Indeed, even in more rigorous study designs, such as randomized controlled trials, findings remain heterogeneous. For example, a secondary analysis of the DO-HEALTH trial demonstrated that participants receiving combined interventions exhibited significantly reduced aging acceleration according to PhenoAge compared with placebo. However, no similar beneficial effects were observed using other epigenetic clocks such as GrimAge, GrimAge2, or DunedinPACE.^47^ This highlights how different BA biomarkers may yield divergent results, likely due to differences in their underlying components, calculation algorithms, or the biological aging processes they reflect—many of which remain incompletely understood.^4^ Therefore, more definitive conclusions on medication effects await a clearer understanding of aging and its biomarkers. Regarding function outcomes, we found that PPIs were associated with subtle improvements in cognitive function, as measured by the MMSE. However, the magnitude of these improvements—0.17 for MMSE (scale of 30)—is too small to be considered substantial. No associations with functional outcomes were observed for the other drug categories. One explanation for the lack of significant functional improvements is the complex nature of aging. Changes at the phenotypic level, such as in body composition, neuroplasticity, or homeostasis, may reflect underlying biological aging processes that are not immediately detectable through physical or cognitive performance.^6^ Also, the translation to functional outcomes, such as physical and cognitive function, is influenced by factors such as diseases, trauma, social engagement, and overall health.^48^ Even if a drug favorably affects phenotypic aging, these other factors may obscure improvements in function.

While this study has several notable strengths—including advanced longitudinal measurements of aging phenotypes, a substantial sample size with a mean follow-up of 9 years, and the application of the cGEE model to control for time-invariant individual factors—several limitations should be acknowledged. First, because medications are often prescribed to manage disease onset or progression, it can be challenging to fully disentangle the effects of the underlying diseases from the effects of the drugs on aging. Many diseases are believed to accelerate aging, potentially biasing the association estimates toward increased aging. In this exploratory study, we focused on drugs associated with decreases in PA markers and controlled for the presence of these indication diseases in our fully adjusted models. Additionally, the associations observed in our study may reflect drug effects in the context of specific conditions, and further investigation is needed to understand their effects in other contexts, such as in healthy individuals. Second, lifestyle modifications following disease diagnosis—whether through self-discipline or medical advice—could influence biological aging and confound the observed relationships between medication use and PA markers. While we accounted for smoking, alcohol drinking, and exercise as indicators of lifestyle changes, residual confounding from unmeasured factors such as diet or sleep patterns may still influence the associations. Third, the BLSA sample consists predominantly of high-functioning, healthy, participants with above-average education levels, limiting the generalizability of our findings.^49^ Additionally, the cGEE model focuses on within-individual changes, meaning the estimates reflect shifts in PA markers when an individual is using versus not using a particular drug category. While this approach provides more accurate estimates by controlling for time-invariant factors, it may also render the results more sample-dependent.^14^ Further studies in other populations are needed to validate our findings. Finally, we used a significance threshold of *P*-value < .05 in this exploratory study, aiming to screen drugs and generate hypotheses about those that may protect against human aging processes. It is important to note the risk of false positives in our findings due to not correcting for multiple association testing. Taken together, these limitations suggest that our findings should be interpreted with caution and viewed as hypothesis-generating, based on the screening of commonly used medications using observational data. They should not be considered as evidence supporting the immediate use of these medications for anti-aging purposes before more rigorous validation through preclinical and clinical studies.

In conclusion, we developed PA markers across 4 key phenotypic domains—body composition, energetics, homeostatic mechanisms, and neuroplasticity/neurodegeneration—and identified 5 drug categories—vitamin D and analogues, PPIs, bisphosphonates, thyroid hormones, and thiazides—that were associated with reductions in one or more of these biomarkers. These findings suggest potential protective effects of these drugs on aging processes in older adults. However, due to the exploratory nature of this study, further research is needed to validate these results, particularly with data from other populations.

## Supplementary Material

glaf128_Supplementary_Data
