# Supplementary material for: Longitudinal associations between medication use and phenotypic aging: insights from the Baltimore longitudinal study of aging
Source: J Gerontol A Biol Sci Med Sci. 2025 Jun 11;80(8):glaf128. doi: 10.1093/gerona/glaf128 (PMC12756985; doi:10.1093/gerona/glaf128)
Supplement: glaf128_Supplementary_Data [file glaf128_supplementary_data.docx]

**Supplementary Table 1. Association between 27 drug categories and markers across four phenotypic domains in the screening analysis, adjusted for chronological age.** N.eff refers to the effective number of users who switched their use of a drug category (e.g., from using to stopping), contributing to the cross-over estimation of drug effects. N represents the total number of participants included in the analysis.

| **Exposure** | | **Outcome  (BA biomaker domain)** | **N.eff/N** | **Beta (95%CI)** | **Nominal P-value** | **Adjusted in Model 2** |
| --- | --- | --- | --- | --- | --- | --- |
| **ATC codes** | **Drug category** |  |  |  |  |  |
| B01AC | Platelet aggregation inhibitors excl. heparin | Body compositions | 409/959 | -0.16 (-0.77, 0.45) | 0.61 | Yes |
| B01AC | Platelet aggregation inhibitors excl. heparin | Energetics | 199/592 | 0.51 (-0.34, 1.37) | 0.24 | Yes |
| B01AC | Platelet aggregation inhibitors excl. heparin | Homeostatic mechanisms | 220/600 | 1.43 (0.15, 2.71) | 0.028 | Yes |
| B01AC | Platelet aggregation inhibitors excl. heparin | Neuroplasticity/neurodegeneration | 137/437 | -0.23 (-0.69, 0.23) | 0.33 | Yes |
| N02BA | Salicylic acid and derivatives | Body compositions | 411/959 | -0.15 (-0.75, 0.45) | 0.63 | Yes |
| N02BA | Salicylic acid and derivatives | Energetics | 202/592 | 0.43 (-0.41, 1.28) | 0.32 | Yes |
| N02BA | Salicylic acid and derivatives | Homeostatic mechanisms | 221/600 | 1.64 (0.36, 2.91) | 0.012 | Yes |
| N02BA | Salicylic acid and derivatives | Neuroplasticity/neurodegeneration | 141/437 | -0.13 (-0.59, 0.32) | 0.57 | Yes |
| A11CC | Vitamin D and analogues | Body compositions | 479/959 | -0.62 (-1.22, -0.01) | 0.045 | Yes |
| A11CC | Vitamin D and analogues | Energetics | 251/592 | 1.11 (0.23, 1.99) | 0.014 | Yes |
| A11CC | Vitamin D and analogues | Homeostatic mechanisms | 262/600 | 0.86 (-0.26, 1.98) | 0.13 | Yes |
| A11CC | Vitamin D and analogues | Neuroplasticity/neurodegeneration | 180/437 | -0.15 (-0.52, 0.21) | 0.42 | Yes |
| C10AA | HMG CoA reductase inhibitors | Body compositions | 272/959 | -0.27 (-1.10, 0.57) | 0.54 | Yes |
| C10AA | HMG CoA reductase inhibitors | Energetics | 125/592 | 0.21 (-1.11, 1.53) | 0.75 | Yes |
| C10AA | HMG CoA reductase inhibitors | Homeostatic mechanisms | 130/600 | 2.18 (0.30, 4.07) | 0.023 | Yes |
| C10AA | HMG CoA reductase inhibitors | Neuroplasticity/neurodegeneration | 76/437 | -0.25 (-0.90, 0.41) | 0.46 | Yes |
| B03BA | Vitamin B12 (cyanocobalamin and analogues) | Body compositions | 210/959 | -0.08 (-1.00, 0.84) | 0.87 | Yes |
| B03BA | Vitamin B12 (cyanocobalamin and analogues) | Energetics | 120/592 | -0.83 (-1.90, 0.25) | 0.13 | Yes |
| B03BA | Vitamin B12 (cyanocobalamin and analogues) | Homeostatic mechanisms | 107/600 | 0.35 (-1.49, 2.20) | 0.71 | Yes |
| B03BA | Vitamin B12 (cyanocobalamin and analogues) | Neuroplasticity/neurodegeneration | 65/437 | 1.18 (0.45, 1.90) | 0.0014 | Yes |
| C07AB | Beta blocking agents, selective | Body compositions | 150/959 | -0.63 (-1.61, 0.35) | 0.21 | Yes |
| C07AB | Beta blocking agents, selective | Energetics | 68/592 | 2.27 (0.62, 3.92) | 0.0069 | Yes |
| C07AB | Beta blocking agents, selective | Homeostatic mechanisms | 74/600 | 1.03 (-1.24, 3.30) | 0.37 | Yes |
| C07AB | Beta blocking agents, selective | Neuroplasticity/neurodegeneration | 35/437 | 0.22 (-0.82, 1.25) | 0.68 | Yes |
| C09AA | ACE inhibitors, plain | Body compositions | 141/959 | -0.24 (-1.29, 0.81) | 0.65 | Yes |
| C09AA | ACE inhibitors, plain | Energetics | 48/592 | -0.33 (-2.34, 1.68) | 0.75 | Yes |
| C09AA | ACE inhibitors, plain | Homeostatic mechanisms | 52/600 | -0.39 (-2.47, 1.70) | 0.72 | Yes |
| C09AA | ACE inhibitors, plain | Neuroplasticity/neurodegeneration | 40/437 | 1.67 (0.90, 2.44) | 0.00002 | Yes |
| A02BC | Proton pump inhibitors | Body compositions | 147/959 | 0.14 (-0.99, 1.27) | 0.81 | Yes |
| A02BC | Proton pump inhibitors | Energetics | 58/592 | 0.95 (-0.88, 2.78) | 0.31 | Yes |
| A02BC | Proton pump inhibitors | Homeostatic mechanisms | 73/600 | 0.36 (-1.76, 2.49) | 0.74 | Yes |
| A02BC | Proton pump inhibitors | Neuroplasticity/neurodegeneration | 43/437 | -1.32 (-2.36, -0.28) | 0.013 | Yes |
| M05BA | Bisphosphonates | Body compositions | 155/959 | -0.64 (-1.72, 0.44) | 0.25 | Yes |
| M05BA | Bisphosphonates | Energetics | 61/592 | -2.06 (-3.98, -0.14) | 0.035 | Yes |
| M05BA | Bisphosphonates | Homeostatic mechanisms | 85/600 | -0.59 (-3.03, 1.85) | 0.63 | Yes |
| M05BA | Bisphosphonates | Neuroplasticity/neurodegeneration | 47/437 | -0.22 (-1.07, 0.63) | 0.61 | Yes |
| H03AA | Thyroid hormones | Body compositions | 77/959 | -1.64 (-3.27, -0.01) | 0.049 | Yes |
| H03AA | Thyroid hormones | Energetics | 29/592 | 2.92 (0.32, 5.53) | 0.028 | Yes |
| H03AA | Thyroid hormones | Homeostatic mechanisms | 37/600 | -0.11 (-2.47, 2.24) | 0.93 | Yes |
| H03AA | Thyroid hormones | Neuroplasticity/neurodegeneration | 25/437 | -1.46 (-2.41, -0.51) | 0.0026 | Yes |
| N02BE | Anilides | Body compositions | 121/959 | 1.07 (-0.10, 2.25) | 0.073 | Yes |
| N02BE | Anilides | Energetics | 47/592 | 1.37 (-0.28, 3.02) | 0.1 | Yes |
| N02BE | Anilides | Homeostatic mechanisms | 59/600 | 3.39 (0.57, 6.22) | 0.018 | Yes |
| N02BE | Anilides | Neuroplasticity/neurodegeneration | 41/437 | 1.18 (0.50, 1.87) | 0.00071 | Yes |
| C03AA | Thiazides, plain | Body compositions | 101/959 | -1.60 (-3.00, -0.20) | 0.025 | Yes |
| C03AA | Thiazides, plain | Energetics | 46/592 | -2.76 (-4.75, -0.77) | 0.0065 | Yes |
| C03AA | Thiazides, plain | Homeostatic mechanisms | 45/600 | -4.23 (-6.94, -1.53) | 0.0022 | Yes |
| C03AA | Thiazides, plain | Neuroplasticity/neurodegeneration | 23/437 | -0.31 (-1.57, 0.95) | 0.63 | Yes |
| A12AA | Calcium | Body compositions | 443/959 | 0.48 (-0.13, 1.09) | 0.12 | No |
| A12AA | Calcium | Energetics | 213/592 | -0.02 (-0.96, 0.93) | 0.97 | No |
| A12AA | Calcium | Homeostatic mechanisms | 247/600 | -0.35 (-1.56, 0.86) | 0.57 | No |
| A12AA | Calcium | Neuroplasticity/neurodegeneration | 150/437 | -0.22 (-0.72, 0.28) | 0.39 | No |
| A02AC | Calcium compounds | Body compositions | 326/959 | 0.16 (-0.58, 0.91) | 0.67 | No |
| A02AC | Calcium compounds | Energetics | 150/592 | 0.22 (-0.94, 1.38) | 0.71 | No |
| A02AC | Calcium compounds | Homeostatic mechanisms | 173/600 | -0.03 (-1.49, 1.43) | 0.97 | No |
| A02AC | Calcium compounds | Neuroplasticity/neurodegeneration | 105/437 | -0.30 (-0.97, 0.36) | 0.37 | No |
| A11GA | Ascorbic acid (vitamin C), plain | Body compositions | 218/959 | -0.08 (-1.00, 0.84) | 0.86 | No |
| A11GA | Ascorbic acid (vitamin C), plain | Energetics | 92/592 | 0.30 (-1.08, 1.69) | 0.67 | No |
| A11GA | Ascorbic acid (vitamin C), plain | Homeostatic mechanisms | 105/600 | -0.91 (-2.80, 0.97) | 0.34 | No |
| A11GA | Ascorbic acid (vitamin C), plain | Neuroplasticity/neurodegeneration | 65/437 | -0.08 (-0.73, 0.57) | 0.81 | No |
| H03CA | Iodine therapy | Body compositions | 254/959 | 0.21 (-0.69, 1.11) | 0.65 | No |
| H03CA | Iodine therapy | Energetics | 120/592 | 0.18 (-1.08, 1.43) | 0.78 | No |
| H03CA | Iodine therapy | Homeostatic mechanisms | 140/600 | 0.96 (-0.61, 2.54) | 0.23 | No |
| H03CA | Iodine therapy | Neuroplasticity/neurodegeneration | 85/437 | -0.16 (-0.81, 0.49) | 0.64 | No |
| M01AE | Propionic acid derivatives | Body compositions | 191/959 | -0.45 (-1.42, 0.53) | 0.37 | No |
| M01AE | Propionic acid derivatives | Energetics | 83/592 | -0.11 (-1.62, 1.40) | 0.88 | No |
| M01AE | Propionic acid derivatives | Homeostatic mechanisms | 104/600 | -0.18 (-1.92, 1.55) | 0.84 | No |
| M01AE | Propionic acid derivatives | Neuroplasticity/neurodegeneration | 55/437 | 0.52 (-0.20, 1.25) | 0.16 | No |
| A06AD | Osmotically acting laxatives | Body compositions | 192/959 | -0.29 (-1.22, 0.65) | 0.55 | No |
| A06AD | Osmotically acting laxatives | Energetics | 85/592 | 0.71 (-0.88, 2.30) | 0.38 | No |
| A06AD | Osmotically acting laxatives | Homeostatic mechanisms | 97/600 | -0.74 (-2.60, 1.12) | 0.43 | No |
| A06AD | Osmotically acting laxatives | Neuroplasticity/neurodegeneration | 64/437 | 0.03 (-0.64, 0.70) | 0.93 | No |
| C09CA | Angiotensin II receptor blockers (ARBs), plain | Body compositions | 145/959 | 0.06 (-0.93, 1.05) | 0.9 | No |
| C09CA | Angiotensin II receptor blockers (ARBs), plain | Energetics | 71/592 | -0.46 (-1.92, 0.99) | 0.53 | No |
| C09CA | Angiotensin II receptor blockers (ARBs), plain | Homeostatic mechanisms | 68/600 | 0.34 (-2.13, 2.80) | 0.79 | No |
| C09CA | Angiotensin II receptor blockers (ARBs), plain | Neuroplasticity/neurodegeneration | 45/437 | -0.30 (-1.00, 0.40) | 0.39 | No |
| C08CA | Dihydropyridine derivatives | Body compositions | 137/959 | 0.76 (-0.56, 2.08) | 0.26 | No |
| C08CA | Dihydropyridine derivatives | Energetics | 61/592 | -1.40 (-3.30, 0.51) | 0.15 | No |
| C08CA | Dihydropyridine derivatives | Homeostatic mechanisms | 70/600 | -0.95 (-3.49, 1.60) | 0.47 | No |
| C08CA | Dihydropyridine derivatives | Neuroplasticity/neurodegeneration | 40/437 | -0.61 (-1.61, 0.38) | 0.23 | No |
| A12CC | Magnesium | Body compositions | 136/959 | -0.15 (-1.28, 0.98) | 0.79 | No |
| A12CC | Magnesium | Energetics | 67/592 | 0.43 (-1.44, 2.31) | 0.65 | No |
| A12CC | Magnesium | Homeostatic mechanisms | 75/600 | -1.62 (-3.79, 0.55) | 0.14 | No |
| A12CC | Magnesium | Neuroplasticity/neurodegeneration | 46/437 | -0.27 (-1.07, 0.53) | 0.51 | No |
| A02AA | Magnesium compounds | Body compositions | 138/959 | -0.32 (-1.48, 0.84) | 0.59 | No |
| A02AA | Magnesium compounds | Energetics | 64/592 | 0.85 (-1.02, 2.73) | 0.37 | No |
| A02AA | Magnesium compounds | Homeostatic mechanisms | 74/600 | -1.28 (-3.51, 0.95) | 0.26 | No |
| A02AA | Magnesium compounds | Neuroplasticity/neurodegeneration | 46/437 | -0.22 (-1.04, 0.59) | 0.59 | No |
| G04CA | Alpha-adrenoreceptor antagonists | Body compositions | 106/959 | -0.29 (-1.32, 0.74) | 0.59 | No |
| G04CA | Alpha-adrenoreceptor antagonists | Energetics | 51/592 | -0.02 (-1.97, 1.93) | 0.98 | No |
| G04CA | Alpha-adrenoreceptor antagonists | Homeostatic mechanisms | 57/600 | 0.46 (-1.65, 2.57) | 0.67 | No |
| G04CA | Alpha-adrenoreceptor antagonists | Neuroplasticity/neurodegeneration | 36/437 | -0.58 (-1.50, 0.35) | 0.22 | No |
| A11EA | Vitamin B-complex, plain | Body compositions | 107/959 | -0.06 (-1.38, 1.26) | 0.93 | No |
| A11EA | Vitamin B-complex, plain | Energetics | 59/592 | 0.11 (-1.88, 2.11) | 0.91 | No |
| A11EA | Vitamin B-complex, plain | Homeostatic mechanisms | 70/600 | 0.11 (-2.18, 2.40) | 0.92 | No |
| A11EA | Vitamin B-complex, plain | Neuroplasticity/neurodegeneration | 37/437 | 0.26 (-0.69, 1.20) | 0.59 | No |
| A12BA | Potassium | Body compositions | 83/959 | -0.53 (-2.09, 1.02) | 0.5 | No |
| A12BA | Potassium | Energetics | 32/592 | 0.02 (-2.32, 2.37) | 0.98 | No |
| A12BA | Potassium | Homeostatic mechanisms | 36/600 | -0.37 (-3.74, 3.01) | 0.83 | No |
| A12BA | Potassium | Neuroplasticity/neurodegeneration | 29/437 | 0.49 (-0.68, 1.67) | 0.41 | No |
| B03BB | Folic acid and derivatives | Body compositions | 79/959 | 0.35 (-1.33, 2.02) | 0.68 | No |
| B03BB | Folic acid and derivatives | Energetics | 25/592 | 0.69 (-1.45, 2.84) | 0.52 | No |
| B03BB | Folic acid and derivatives | Homeostatic mechanisms | 44/600 | 0.80 (-2.23, 3.84) | 0.6 | No |
| B03BB | Folic acid and derivatives | Neuroplasticity/neurodegeneration | 21/437 | -0.59 (-1.94, 0.76) | 0.39 | No |
| A02BA | H2-receptor antagonists | Body compositions | 82/959 | 0.42 (-0.78, 1.63) | 0.49 | No |
| A02BA | H2-receptor antagonists | Energetics | 38/592 | 1.14 (-0.51, 2.78) | 0.17 | No |
| A02BA | H2-receptor antagonists | Homeostatic mechanisms | 40/600 | 1.17 (-1.70, 4.04) | 0.42 | No |
| A02BA | H2-receptor antagonists | Neuroplasticity/neurodegeneration | 32/437 | -0.51 (-1.47, 0.45) | 0.29 | No |

**Supplementary Table 2. Hazard ratio estimates for mortality per a five-year increase in each phenotypic aging marker.** HRs were derived from Cox proportional hazards models, adjusting for age and sex. The analysis utilized the first available non-missing PA value for each participant.

| **Phenotypic aging marker** | **HR (95%CI)** | **P-value** |
| --- | --- | --- |
| Body compositions | 1.08 (1.03,1.13) | 0.0011 |
| Energetics | 1.19 (1.11,1.27) | <0.001 |
| Homeostatic mechanisms | 1.07 (1.01,1.13) | 0.016 |
| Neuroplasticity/neurodegeneration | 1.27 (1.13,1.43) | <0.001 |

**Supplementary Table 3. Associations between drug categories and markers across four phenotypic domains.** Adjustments were made for age, living alone, health insurance status, annual family income, highest education level, current smoking and drinking status, Charlson Comorbidity Index (CCI), other drug categories with a P-value < 0.05 from Model 1, and the presence of indication diseases for the drug categories identified in Model 1, including hypertension, dyslipidemia, heart failure, myocardial infarction, edema, hypothyroidism, vitamin D deficiency, hypocalcemia, osteoporosis, and diseases of the esophagus, stomach, and duodenum. N.eff refers to the effective number of users who switched their use of the drug category (e.g., from use to non-use), contributing to the cross-over estimation of drug effects. N represents the total number of participants included in the analysis.

| **Exposure** | | **Outcome  (BA biomaker domain)** | **Men** | | | **Women** | | | **African American** | | | **White** | | |
| --- | --- | --- | --- | --- | --- | --- | --- | --- | --- | --- | --- | --- | --- | --- |
|  |  |  |  |  |  |  |  |  |  |  |  |  |  |  |
| **ATC codes** | **Drug category** |  | **N.eff/N** | **Beta (95%CI)** | **P-value** | **N.eff/N** | **Beta (95%CI)** | **P-value** | **N.eff/N** | **Beta (95%CI)** | **P-value** | **N.eff/N** | **Beta (95%CI)** | **P-value** |
| A11CC | Vitamin D and analogues | Body compositions | 194/442 | -0.79  (-1.45, -0.12) | 0.021 | 273/505 | -0.70  (-1.64, 0.23) | 0.14 | 127/239 | -1.02  (-2.30, 0.26) | 0.12 | 309/656 | -0.53  (-1.23, 0.18) | 0.14 |
| A02BC | Proton pump inhibitors | Neuroplasticity/neurodegeneration | 18/191 | -1.86  (-3.71, -0.02) | 0.048 | 22/238 | -0.49  (-1.61, 0.63) | 0.39 | 9/102 | -1.65  (-3.59, 0.29) | 0.095 | 29/297 | -0.61  (-1.87, 0.64) | 0.34 |
| M05BA | Bisphosphonates | Energetics | 17/264 | -0.21  (-2.62, 2.20) | 0.86 | 44/313 | -2.56  (-5.01, -0.12) | 0.04 | 13/152 | -2.73  (-7.57, 2.11) | 0.27 | 41/387 | -1.47  (-3.84, 0.91) | 0.23 |
| H03AA | Thyroid hormones | Body compositions | 27/442 | -1.92  (-4.10, 0.26) | 0.084 | 49/505 | -1.73  (-3.72, 0.26) | 0.089 | 14/239 | -2.49  (-6.04, 1.06) | 0.17 | 54/656 | -1.65  (-3.44, 0.14) | 0.07 |
|  |  | Neuroplasticity/neurodegeneration | 7/191 | -1.57  (-4.01, 0.87) | 0.21 | 18/238 | -0.73  (-1.67, 0.21) | 0.13 | 5/102 | 0.24  (-1.56, 2.05) | 0.79 | 17/297 | -1.48  (-2.59, -0.38) | 0.0084 |
| C03AA | Thiazides, plain | Body compositions | 47/442 | -0.49  (-1.85, 0.86) | 0.48 | 54/505 | -2.36  (-4.66, -0.07) | 0.043 | 34/239 | -1.19  (-3.29, 0.90) | 0.26 | 60/656 | -1.47  (-3.03, 0.08) | 0.063 |
|  |  | Energetics | 18/264 | -2.24  (-5.29, 0.82) | 0.15 | 27/313 | -1.74  (-4.08, 0.60) | 0.14 | 18/152 | -4.99  (-8.30, -1.67) | 0.0032 | 23/387 | -1.81  (-4.16, 0.54) | 0.13 |
|  |  | Homeostatic mechanisms | 21/275 | -5.63  (-9.78, -1.49) | 0.0078 | 23/312 | -2.88  (-6.80, 1.05) | 0.15 | 12/126 | -9.71  (-14.26, -5.15) | 0.00003 | 27/423 | -4.33  (-7.29, -1.37) | 0.0041 |

**Supplementary Table 4. Associations between drug categories and cognitive and physical functional outcomes.** The model was adjusted for age, living alone, health insurance status, annual family income, highest education level, current smoking and drinking status, Charlson Comorbidity Index, other drug categories with a P-value < 0.05 from Model 1, and the presence of indication diseases identified in Model 1, including hypertension, dyslipidemia, heart failure, myocardial infarction, edema, hypothyroidism, vitamin D deficiency, hypocalcemia, osteoporosis, and diseases of the esophagus, stomach, and duodenum. N.eff refers to the effective number of users who switched their use of the drug category (e.g., from use to non-use), contributing to the cross-over estimation of drug effects. N represents the total number of participants included in the analysis. Physical function was represented by the Health, Aging and Body Composition short physical performance battery (HABC SPPB), a continuous score derived from four measurements: usual gait speed; time taken to stand up and sit back down five times on an armless chair without assistance; ability to hold three balance-related positions (semi-tandem, full-tandem, and single-leg stands) for up to 30 seconds each; and capacity and time to walk a narrow (20 cm wide) 6-meter course. Global cognitive function was assessed using the Mini-Mental State Examination (MMSE).

| **Exposure** | | **Outcome** | **N.eff/N** | **Beta (95%CI)** | **P-value** |
| --- | --- | --- | --- | --- | --- |
| **ATC codes** | **Drug category** |  |  |  |  |
| A11CC | Vitamin D and analogues | PhenoAge | 385/799 | -0.35 (-0.59, -0.12) | 0.0033 |
|  |  | HABCPPB | 465/917 | 0.03 (-0.00, 0.06) | 0.057 |
|  |  | MMSE | 439/892 | 0.03 (-0.06, 0.13) | 0.51 |
| A02BC | Proton pump inhibitors | PhenoAge | 101/799 | 0.15 (-0.28, 0.58) | 0.4800 |
|  |  | HABCPPB | 135/917 | 0.03 (-0.03, 0.09) | 0.27 |
|  |  | MMSE | 130/892 | 0.17 (0.01, 0.33) | 0.033 |
| M05BA | Bisphosphonates | PhenoAge | 127/799 | -0.14 (-0.53, 0.26) | 0.4900 |
|  |  | HABCPPB | 145/917 | 0.01 (-0.04, 0.07) | 0.67 |
|  |  | MMSE | 141/892 | 0.19 (-0.01, 0.39) | 0.069 |
| H03AA | Thyroid hormones | PhenoAge | 62/799 | 0.12 (-0.45, 0.69) | 0.67 |
|  |  | HABCPPB | 76/917 | -0.02 (-0.11, 0.07) | 0.66 |
|  |  | MMSE | 74/892 | 0.15 (-0.07, 0.38) | 0.19 |
| C03AA | Thiazides, plain | PhenoAge | 62/799 | -0.52 (-1.14, 0.10) | 0.10 |
|  |  | HABCPPB | 93/917 | -0.06 (-0.12, 0.01) | 0.091 |
|  |  | MMSE | 93/892 | 0.04 (-0.19, 0.27) | 0.73 |

**Supplementary Figure 1. Trajectories for measurements included in the estimation of phenotypic aging maker for body composition.** Each dot represents a measurement for one participant at a single observation, while the spaghetti lines illustrate changes between observations for the same participant. The trajectories were estimated separately for men and women using a linear mixed model, with age and sex as fixed effects and individuals as random effects.


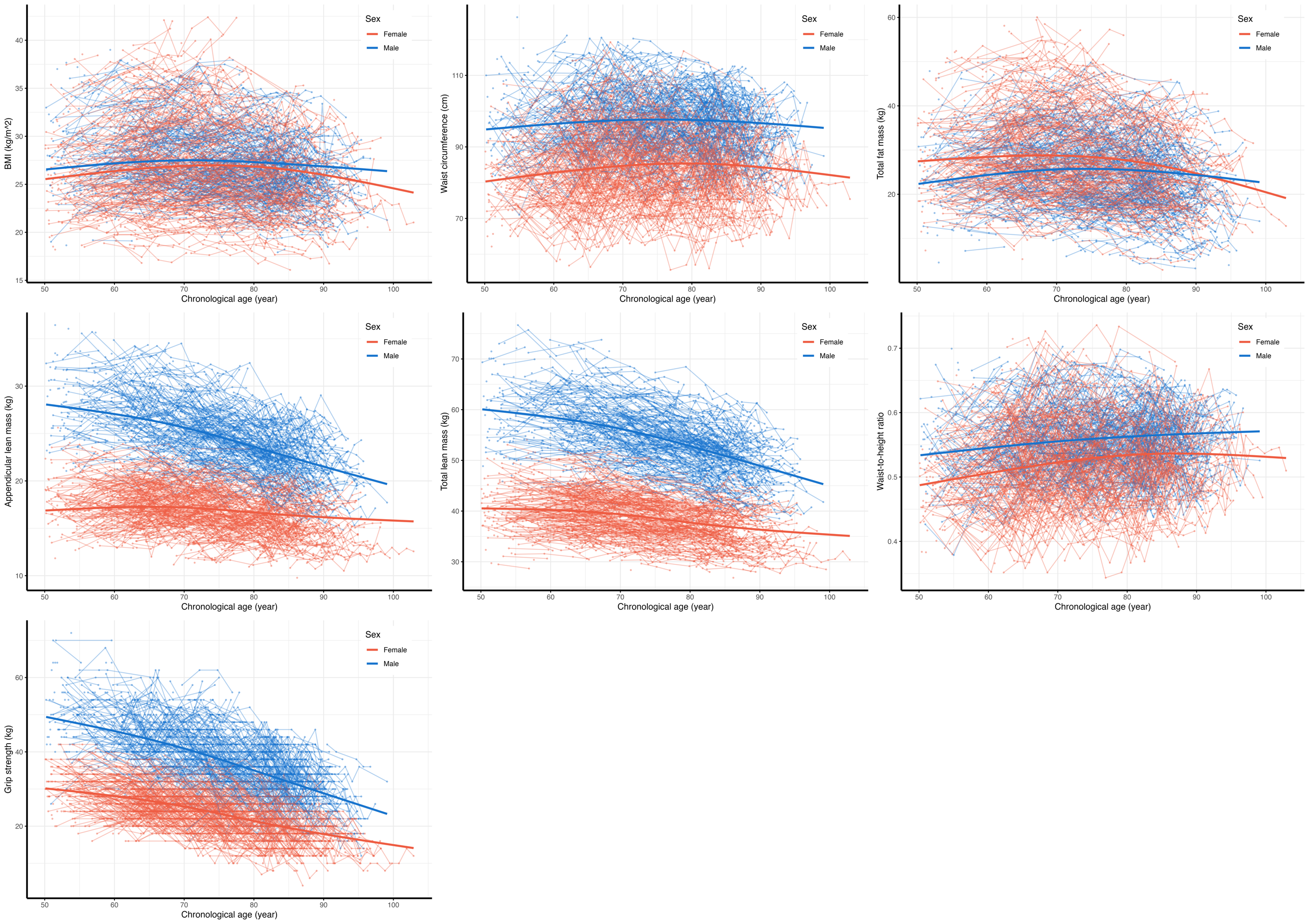


**Supplementary Figure 2. Trajectories for measurements included in the estimation of phenotypic aging maker for energetics.** Each dot represents a measurement for one participant at a single observation, while the spaghetti lines illustrate changes between observations for the same participant. The trajectories were estimated separately for men and women using a linear mixed model, with age and sex as fixed effects and individuals as random effects.


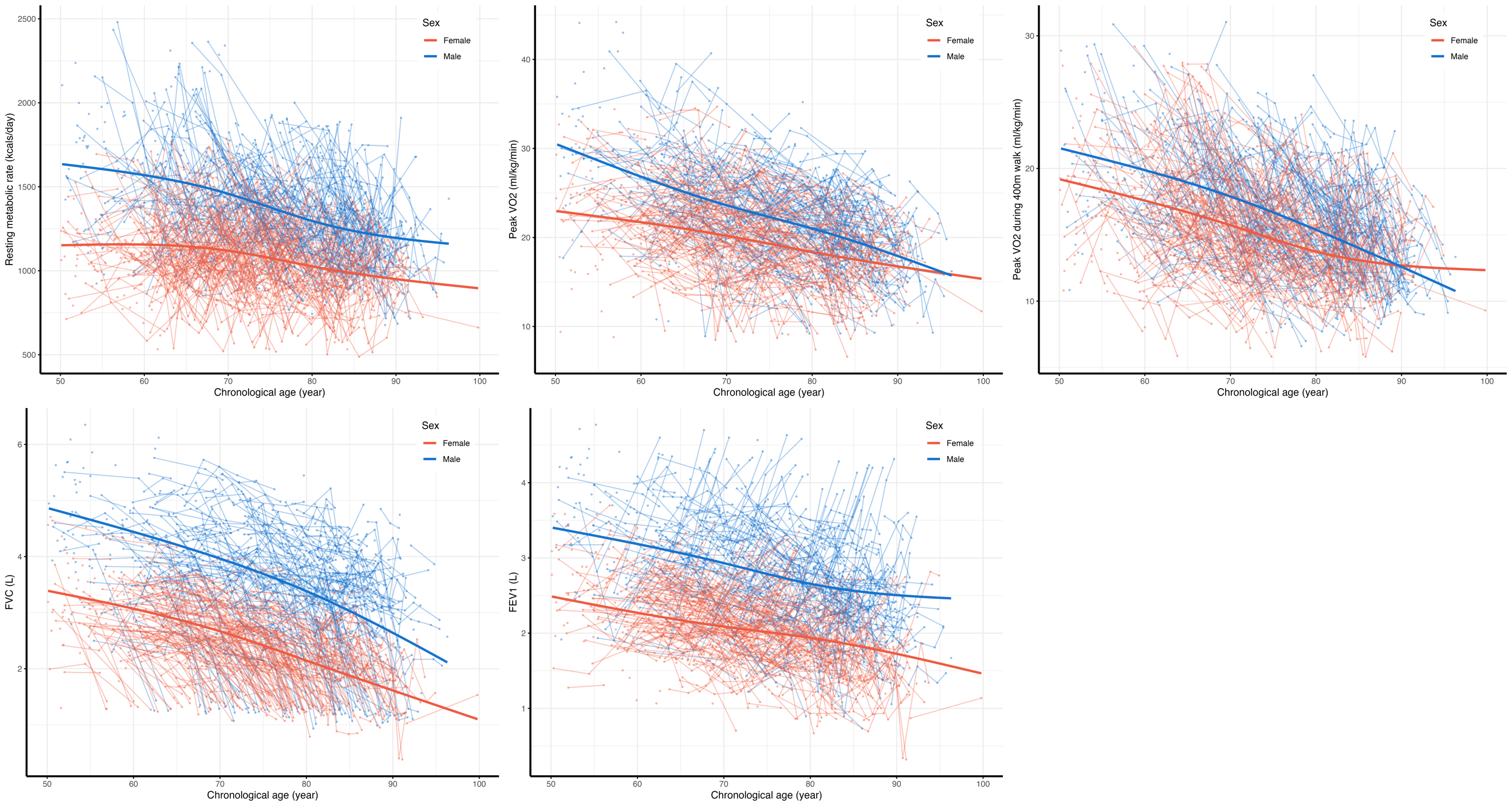


**Supplementary Figure 3. Trajectories for measurements included in the estimation of phenotypic aging maker for homeostatic mechanisms.** Each dot represents a measurement for one participant at a single observation, while the spaghetti lines illustrate changes between observations for the same participant. The trajectories were estimated separately for men and women using a linear mixed model, with age and sex as fixed effects and individuals as random effects.


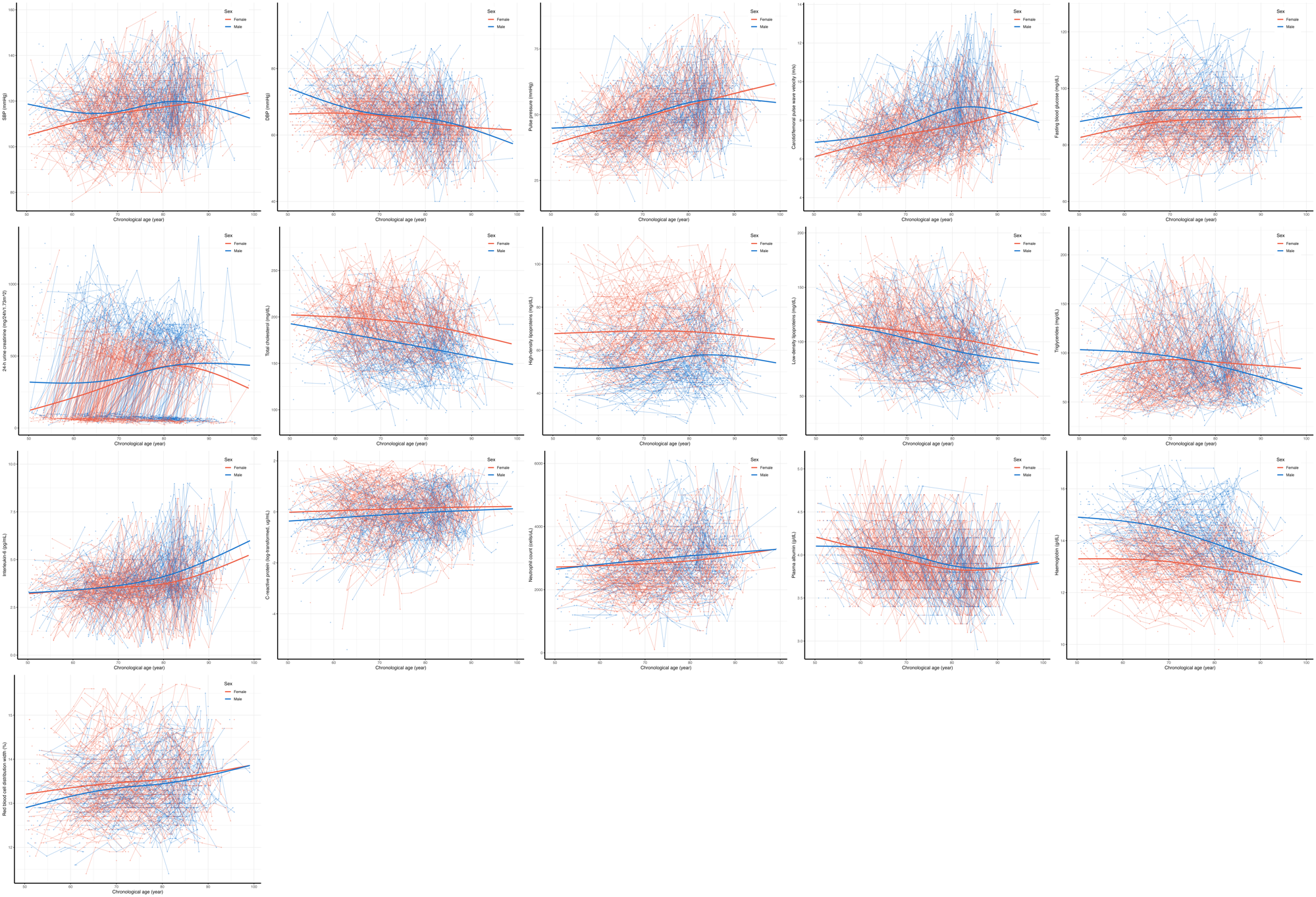


**Supplementary Figure 4. Trajectories for measurements included in the estimation of phenotypic aging maker for neuroplasticity/neurodegeneration.** Each dot represents a measurement for one participant at a single observation, while the spaghetti lines illustrate changes between observations for the same participant. The trajectories were estimated separately for men and women using a linear mixed model, with age and sex as fixed effects and individuals as random effects.


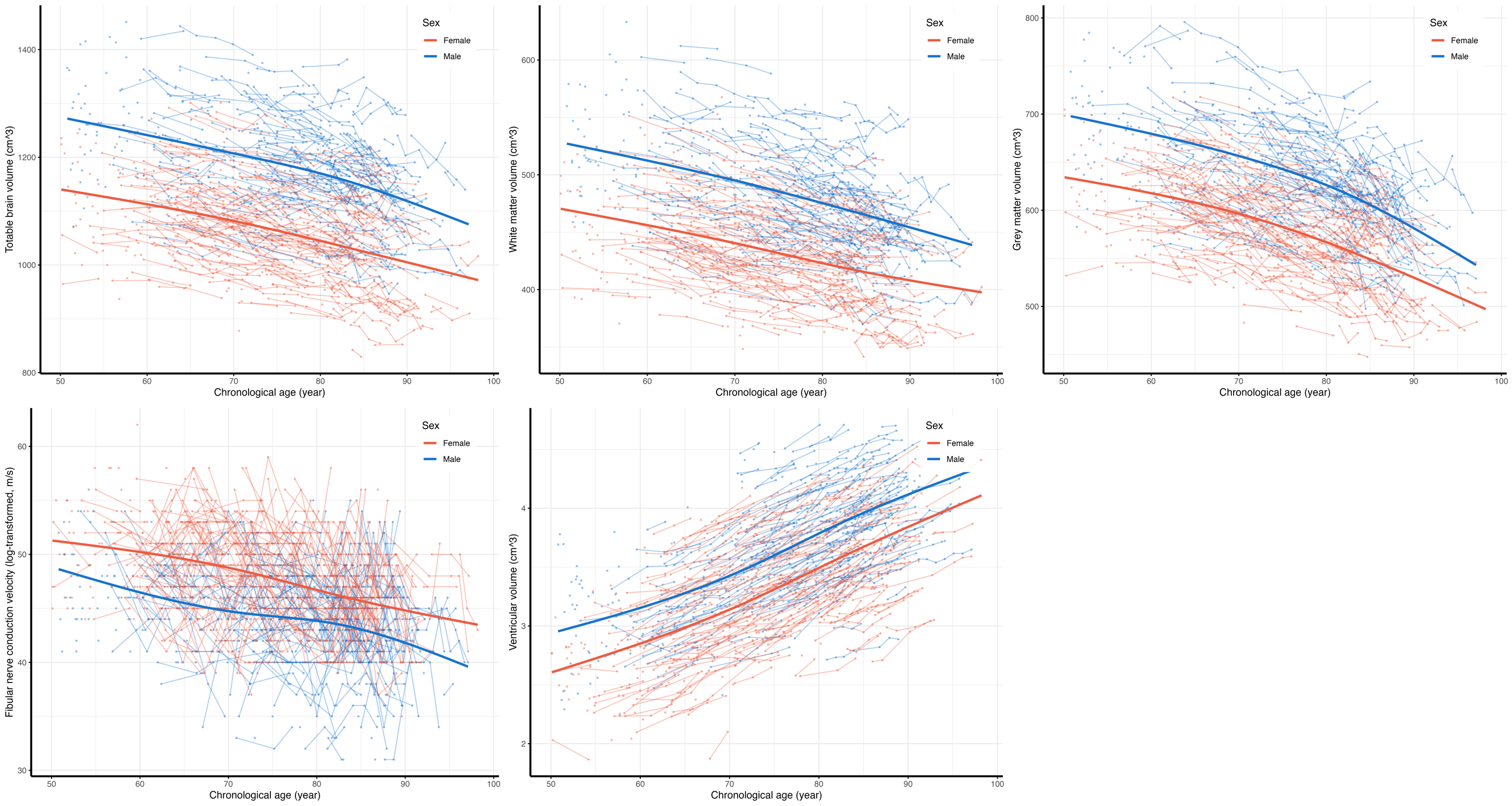


**Supplementary Figure 5. Trajectories for measurements included in the estimation of PhenoAge.** Each dot represents a measurement for one participant at a single observation, while the spaghetti lines illustrate changes between observations for the same participant. The trajectories were estimated separately for men and women using a linear mixed model, with age and sex as fixed effects and individuals as random effects.

**
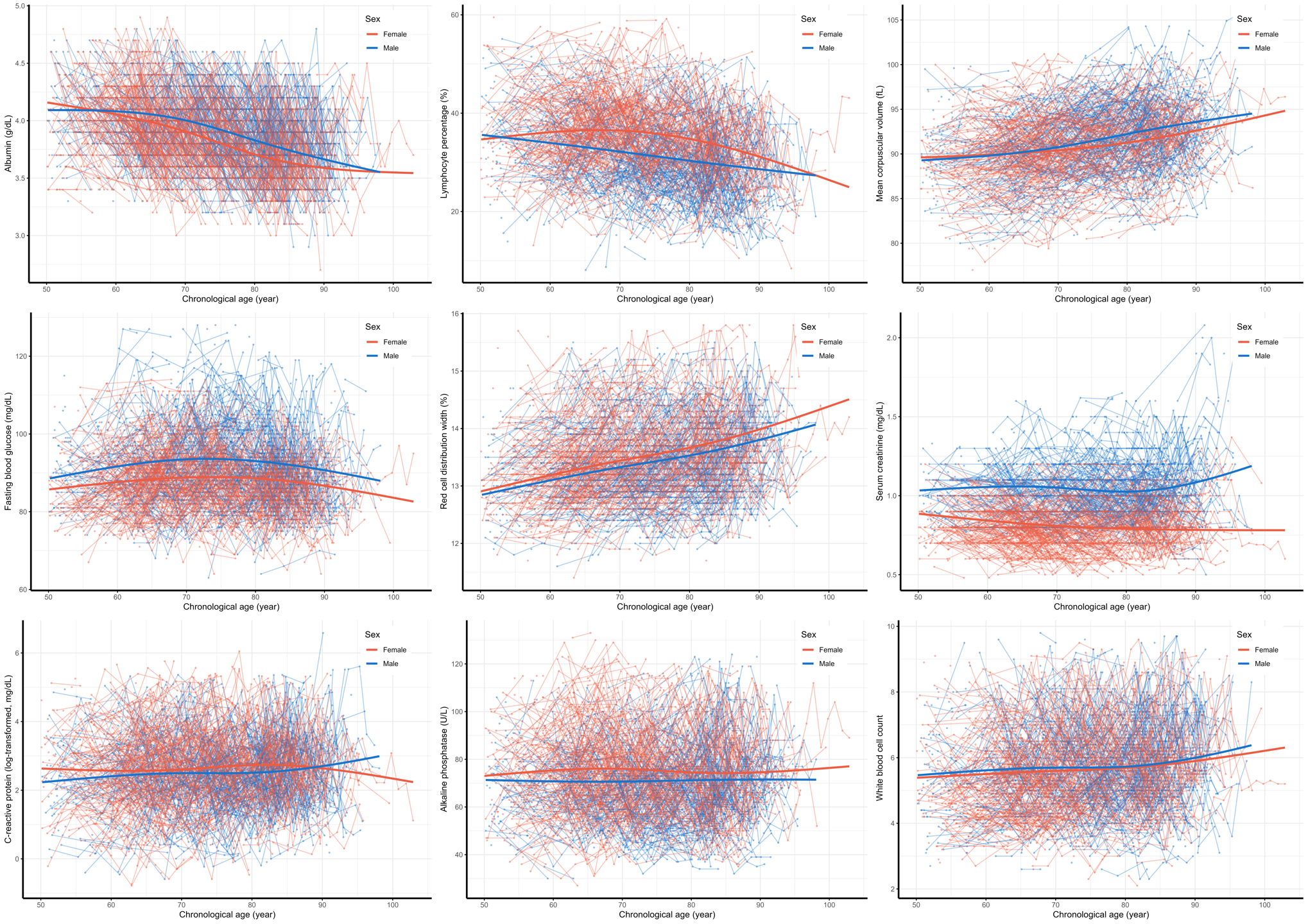
**

**Supplementary Figure 6. Trajectories of PhenoAge over age.** Each dot represents the PhenoAge for a single observation, while the spaghetti lines illustrate changes between observations for the same participant. The trajectories were estimated separately for men and women using a linear mixed model, with an interaction term of age and sex as the fixed effect, and individuals as the random effect. The r represents the correlation coefficient, estimated using the repeated measures correlation method. The correlation coefficients are provided for the overall sample (r), men (r_m), and women (r_w). The HR_mortality represents the hazard ratio per a five-year increase in the PA marker from a Cox model, adjusted for age and sex. The first available observation for each participant with a non-missing PhenoAge was used in the survival analysis.

**
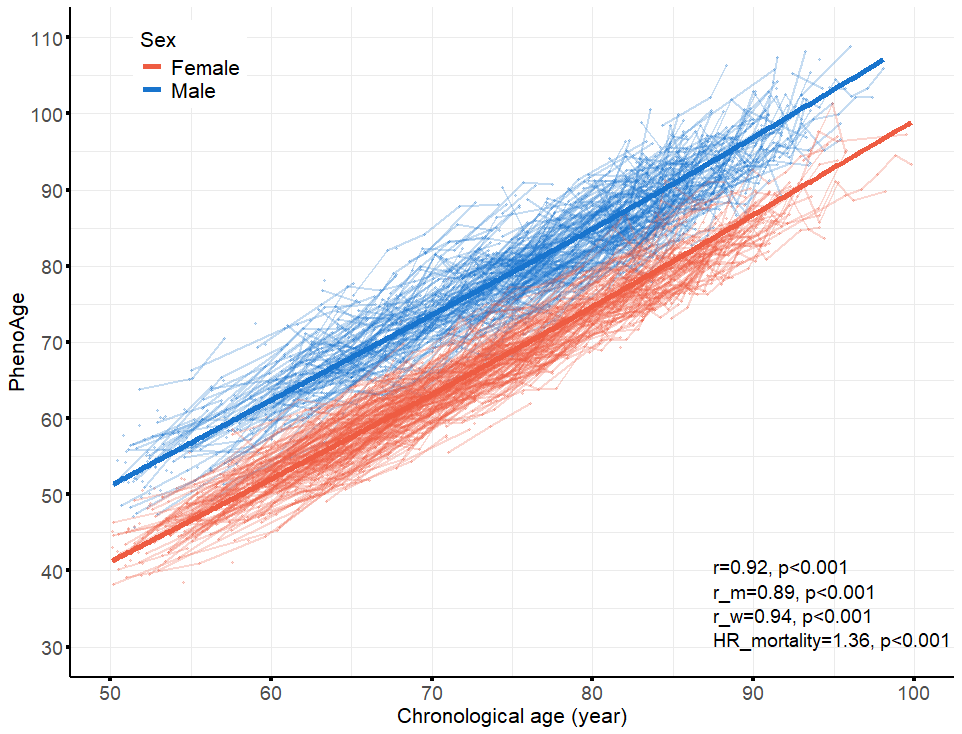
**

**Supplementary Figure 7. Pairwise correlations between phenotypic aging makers for body composition, energetics, homeostatic mechanisms, and neuroplasticity/neurodegeneration.** Panel a displays the pairwise correlations between the biological age biomarkers. Panel b shows the correlations for the residuals of these biomarkers after regressing out the effects of chronological age and sex. The correlations were estimated using the repeated measures correlation method. The residuals of the biological age biomarkers were obtained using a linear mixed model, with sex and age as fixed effects and individuals as random effects.** P-value for correlation < 0.05/6

**
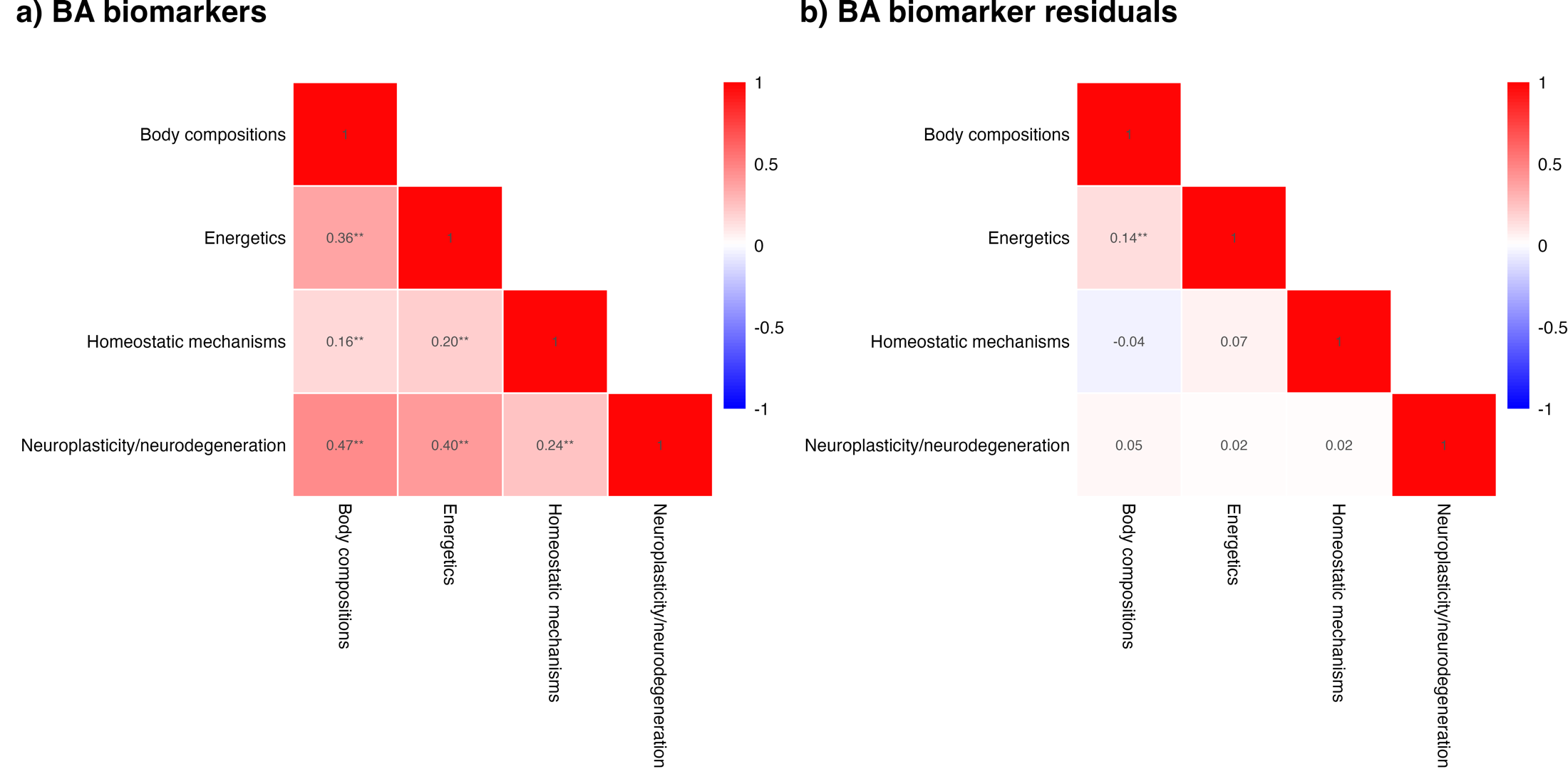
**

**Supplementary Methods – Modified Klemera-Doubal Method (KDM) for Biological Age Estimation**

#' Modified KDM Algorithm for Biological Age Estimation

#'

#' This function implements a modified version of the Klemera and Doubal (KDM) method

#' to estimate biological age using physiological biomarkers through cross-validated

#' principal component analysis (PCA) and linear modeling.

#'

#' @param data A data frame containing longitudinal observations with columns:

#' idno (individual ID), visit (visit identifier), sex, age, and biomarker measurements.

#' @param measures A character vector of biomarker column names to use in the model.

#' @param num_folds Number of cross-validation folds (default = 10).

#' @param num_iterations Number of iterations for repeated cross-validation (default = 10).

#' @param seed Random seed for reproducibility (default = 123).

#'

#' @return A data frame with biological age estimates (BAe) and delta values.

#'

#' Modified KDM Algorithm for Biological Age Estimation

#'

#' This function implements a modified Klemera-Doubal method using PCA and

#' repeated cross-validation without separate helper functions.

#' Modified KDM Biological Age Estimation with Repeated Cross-Validation

#'

#' Estimates biological age using a modified Klemera-Doubal method with PCA

#' and repeated cross-validation. Handles longitudinal data with sex stratification.

#'

#' @param data Data frame containing:

#' - idno: Participant ID

#' - visit: Visit identifier

#' - sex: Biological sex (Male/Female)

#' - age: Chronological age

#' - [biomarker columns]

#' @param measures Character vector of biomarker column names

#' @param num_folds Number of CV folds (default = 10)

#' @param num_iterations Number of CV repetitions (default = 10)

#' @param seed Random seed (default = 123)

#'

#' @return Data frame with:

#' - idno: Participant ID

#' - visit: Visit identifier

#' - BAe_mean: Mean estimated biological age

#' - BA_delta_mean: Mean age acceleration

#'

#' @importFrom caret createFolds

#' @importFrom dplyr select mutate filter group_by ungroup starts_with

#' @importFrom stats prcomp lm predict

#' @export

kdm_cross_iteration_noCA <- function(data, measures, num_folds = 10, num_iterations = 10, seed = 123) {

# ---------------------------

# 1. Initial Setup

# ---------------------------

set.seed(seed)

library(caret)

library(dplyr)

# Input validation

required_cols <- c("idno", "visit", "sex", "age")

if (!all(required_cols %in% colnames(data))) {

stop("Missing required columns: ", paste(setdiff(required_cols, colnames(data)), collapse = ", "))

}

# ---------------------------

# 2. Data Preparation

# ---------------------------

processed_data <- data %>%

dplyr::select(all_of(required_cols), all_of(measures)) %>%

mutate(

idno = as.character(idno),

visit = as.character(visit)

)

# ---------------------------

# 3. Core Algorithm

# ---------------------------

calculate_kdm <- function(df) {

ids <- unique(df$idno)

all_iterations <- vector("list", num_iterations)

for (iter in seq_len(num_iterations)) {

folds <- caret::createFolds(ids, k = num_folds, list = TRUE)

fold_results <- vector("list", num_folds)

# ---------------------------

# 3a. Cross-Validation Fold

# ---------------------------

for (fold in seq_along(folds)) {

# Data partitioning

test_ids <- ids[folds[[fold]]]

train_data <- df %>% filter(!idno %in% test_ids)

test_data <- df %>% filter(idno %in% test_ids)

# ---------------------------

# 3b. Training Phase

# ---------------------------

# Baseline selection and scaling

train_base <- train_data %>%

group_by(idno) %>%

filter(visit == min(visit)) %>%

ungroup()

# Scale training baseline

for (bm in measures) {

train_mean <- mean(train_base[[bm]], na.rm = TRUE)

train_sd <- sd(train_base[[bm]], na.rm = TRUE)

# Scale training data

train_base[[bm]] <- (train_base[[bm]] - train_mean) / train_sd

# Scale test data using training parameters

test_data[[bm]] <- (test_data[[bm]] - train_mean) / train_sd

}

# Now proceed with PCA and KDM calculations using:

# - train_base (scaled training baseline)

# - test_data (scaled test data)

# PCA transformation

pca_model <- prcomp(train_base[, measures], center = FALSE, scale. = FALSE)

train_pcs <- cbind(train_base, pca_model$x)

test_pcs <- cbind(test_data[, required_cols], as.matrix(test_data[, measures]) %*% pca_model$rotation)

# ---------------------------

# 3c. KDM Calculation

# ---------------------------

# Fit age models for principal components

pc_cols <- grep("^PC", colnames(train_pcs), value = TRUE)

model_params <- lapply(pc_cols, function(pc) {

model <- lm(paste(pc, "~ age"), data = train_pcs)

list(

coef = coef(model),

rmse = summary(model)$sigma,

rsq = summary(model)$r.squared

)

})

names(model_params) <- pc_cols

# Calculate KDM weights

weight_df <- data.frame(

PC = pc_cols,

B_age = sapply(model_params, `[[`, "coef")["age", ],

RMSE = sapply(model_params, `[[`, "rmse"),

rsq = sapply(model_params, `[[`, "rsq")

) %>% mutate(

r1 = abs((B_age / RMSE) * sqrt(rsq)),

r2 = abs(B_age / RMSE),

n2 = (B_age / RMSE)^2

)

# ---------------------------

# 3d. Biological Age Estimation

# ---------------------------

test_matrix <- as.matrix(test_pcs[, pc_cols])

predicted_vals <- sapply(pc_cols, function(pc) {

predict(lm(paste(pc, "~ age"), data = train_pcs), newdata = test_pcs)

})

ba_results <- test_pcs %>%

mutate(

BA_delta = rowSums(

(test_matrix - predicted_vals) * (weight_df$B_age / (weight_df$RMSE^2)),

na.rm = TRUE

) / sum(weight_df$n2),

BAe = age + BA_delta

) %>%

select(idno, visit, BAe, BA_delta)

fold_results[[fold]] <- ba_results

}

all_iterations[[iter]] <- bind_rows(fold_results)

}

# ---------------------------

# 4. Result Aggregation

# ---------------------------

bind_rows(all_iterations) %>%

group_by(idno, visit) %>%

summarize(

BAe_mean = mean(BAe, na.rm = TRUE),

BA_delta_mean = mean(BA_delta, na.rm = TRUE),

.groups = "drop"

)

}

# ---------------------------

# 5. Sex-Stratified Analysis

# ---------------------------

bind_rows(

calculate_kdm(filter(processed_data, sex == "Male")),

calculate_kdm(filter(processed_data, sex == "Female"))

)

}
